# Differential virulence and immune recognition of *Klebsiella pneumoniae* O-antigen subtypes O2α and O2β

**DOI:** 10.1128/iai.00538-25

**Published:** 2025-11-28

**Authors:** Paeton L. Wantuch, Lloyd S. Robinson, Cory J. Knoot, Christian M. Harding, David A. Rosen

**Affiliations:** 1 Department of Pediatrics, Division of Infectious Diseases, Washington University School of Medicine 12275 , St. Louis, Missouri, USA; 2 Omniose, St. Louis, Missouri, USA; 3 Department of Molecular Microbiology, Washington University School of Medicine 12275 , St. Louis, Missouri, USA; University of California San Diego School of Medicine, La Jolla, California, USA

**Keywords:** *Klebsiella pneumoniae*, vaccine, O-antigen polysaccharide, bioconjugation, pulmonary infections

## Abstract

*Klebsiella pneumoniae* infections are sharply on the rise among at-risk populations. *K. pneumoniae* has nine serogroups of O-antigens. Recently, additional O-antigen subtypes within these serogroups have been identified; the contributions of these subtypes to pathogenic fitness and their immunogenicity, functional antibody responses, and cross-reactivity are unknown. We investigated how the addition of the single-branched galactose in O-antigen subtype O2b compared to O2a alters its virulence and host immune responses. We deleted the *gmlABC* region of an O2b strain of *K. pneumoniae*, converting it to an otherwise isogenic O2a strain. Complementation of this mutant allowed us to identify the specific genes responsible for the addition of the single branched galactose of O2b. Experiments using the O2a mutant and its parent O2b strain confirmed similar phenotypic expression of virulence factors beyond the O-antigen. Well-established murine models of pneumonia were used to determine the pulmonary fitness of the strains and assess the host innate immune responses. Complement-mediated killing assays suggested differences in susceptibility to innate immune defenses, with the O2a mutant being more susceptible to serum killing. Lastly, using polysaccharide-protein bioconjugate vaccines against these specific O-antigen subtypes, we determined that only partial cross-reactivity and protection are elicited. These studies advance our understanding of the immune response to *K. pneumoniae* O-antigens by defining a fitness advantage of O2b compared to O2a and informing vaccine design to combat this drug-resistant pathogen.

## INTRODUCTION

*Klebsiella pneumoniae* is a Gram-negative opportunistic pathogen capable of causing a variety of infection types across various hosts. It often causes hospital-acquired invasive infection in immunocompromised hosts; however, some pathotypes can cause disease in otherwise healthy individuals. Over the course of the last few decades, *K. pneumoniae* has gained an assortment of antibiotic resistance determinants and represents the “K” in the ESKAPE group of antibiotic-resistant pathogens (1). The widespread emergence of multidrug-resistant clones, including those harboring *K. pneumoniae* carbapenemases (KPCs) or extended-spectrum β-lactamases (ESBLs), has led to increasing use of last-resort antibiotics, and even these drugs are losing efficacy (2). *K. pneumoniae* is among the most prevalent pathogens associated with overall global deaths and deaths associated with antimicrobial resistance (3).

Given the decreasing susceptibility of *K. pneumoniae* to available antibiotics and lack of other treatment options, an alternative therapeutic is desperately needed. Immunization to prevent *K. pneumoniae* disease is an attractive approach. Currently, there is no licensed vaccine available. One antigenic target for a *K. pneumoniae* vaccine development has been the O-antigen polysaccharide (OPS) of lipopolysaccharide (LPS). The OPS is the outermost polysaccharide in the LPS structure, making it an accessible target. Furthermore, the OPS of *K. pneumoniae* exhibits limited heterogeneity compared to other polysaccharides such as the capsular polysaccharide. A recent study seeking to consolidate the *K. pneumoniae* OPS groups has demonstrated nine different serogroups (3). Importantly, several of the more complex serogroups contain structurally related subtypes that produce distinct antigenic epitopes (3).

The O2 serogroup of *K. pneumoniae* is one of the most common serogroups associated with antimicrobial-resistant isolates and is composed of multiple subtypes. The two most prominent subtypes within this serogroup are O2α and O2β (formerly referred to as O2a and O2afg) (3). All subtypes within the O2 serogroup share the same backbone composed of a disaccharide repeating unit O2α [→3-β-D-Gal*f*-(1→3)- α-D-Gal*p*-1→], with the OPS of O2β isolates further modified by an α1,4-linked-DGal*p* residue (3, 4). The addition of this extra galactose residue is facilitated by three additional genes, *gmlABC*, within the O2 biosynthesis operon, which are predicted to encode a flippase, synthetase, and glycosyltransferase, respectively (4). Interestingly, a recent study examining over 50,000 isolates of *K. pneumoniae* found that 26.2% of isolates expressed OPS of the O2β type, a higher percentage than any other OPS type (3). Further, another study examining over 700 isolates of *K. pneumoniae* found that 50% of carbapenem-resistant isolates expressed an O2 OPS and, of those, 49% belonged to the clonal group ST258 (5). It has previously been demonstrated that the vast majority of ST258 isolates express the O2β subtype (6). Finally, an investigation examining over 500 *K*. *pneumoniae* isolates found that 67% of them were O2 (7) and, of those, 42% were O2β (4). Together, these studies demonstrate the seroepidemiological importance of the O2β antigen among *K. pneumoniae* clinical isolates, and its strong representation suggests a potential role in fitness.

Given the large proportion of *K. pneumoniae* isolates expressing the O2 serotype and the association of these isolates with antibiotic resistance, we aimed to investigate differences between the two major O2 subtypes, O2α and O2β. Several investigations have explored this topic previously (4, 6, 8), with the majority assessing differences using an O2α expressing strain and providing the *gmlABC* genes *in trans* to produce O2β. However, these studies did not address the growing question of cross-reactivity and cross-protection among different OPS subtypes in the same serogroup. Our previous data suggest that not all subtypes within a serogroup strongly cross-react with each other (9, 10). Further, serologic cross-reactivity does not necessarily equate to *in vivo* cross-protection, which requires additional investigation.

Herein, we assessed differences between O2α and O2β using isogenic isolates differing only in their O-antigen. We removed the *gmlABC* gene cluster from an O2β expressing strain to successfully convert it to an O2α expressing strain. These strains of *K. pneumoniae*, and their complements, were used to explore differences between these two OPS subtypes and test for cross-protection using conjugate vaccines targeting each subtype. Understanding the differences between various subtypes of *K. pneumoniae* OPS is crucial to our understanding of virulence and ultimately will inform vaccine design.

## MATERIALS AND METHODS

### Bacterial strains and growth conditions

The classical *K. pneumoniae* clinical lung isolate KR74 was previously described (11). This strain was obtained from Barnes-Jewish Hospital in St. Louis, MO, and collection was approved by Washington University in St. Louis Institutional Review Board (IRB protocol 201409121). A complete list of all strains used in this study, including mutants, is shown in Table 1. All bacterial strains were grown statically at 37°C for 16 h in Luria-Bertani broth. After growth cultures were centrifuged at 8,000 × *g* for 10 min, the pellet was resuspended in sterile PBS to desired concentration. The desired inoculum concentration was based on optical density (OD) at 600 nm. The inocula were confirmed by serial dilution and plating.

**TABLE 1:** Strains and plasmids used in this study

Strain name	Description	Reference
KR74	Wild-type strain: O2β expressing	(11)
*gmlABC*	KR74 with *gmlABC* genes knocked out: O2α expressing	This study
D*gmlABC* /pACT3	Empty vector control: O2α expressing	This study
D*gmlABC*/p*gmlA*	*gmlA* complemented back on pACT3 plasmid	This study
D*gmlABC*/p*gmlB*	*gmlB* complemented back on pACT3 plasmid	This study
D*gmlABC*/p*gmlC*	*gmlC* complemented back on pACT3 plasmid	This study
D*gmlABC*/p*gmlBC*	*gmlBC* complemented back on pACT3 plasmid	This study
D*gmlABC*/p*gmlABC*	*gmlABC* complemented back on pACT3 plasmid: O2β expressing	This study
TOP52	Classical *K. pneumoniae* isolate used as reference	(12)
pKD4	Plasmid used in λ Red recombinase method containing kanamycin cassette	(13)
pKD46s	Plasmid used in λ Red recombinase method containing spectinomycin resistance	(13)

### Construction of mutants and complementation plasmids

A modified l Red recombinase protocol was utilized to construct the Δ*gmlABC* mutant using pKD46 as previously described (13). All primers used in this study are shown in Table S1. Mutants were confirmed by sequencing. Complemented strains were constructed using standard restriction enzymes and ligation protocol. Briefly, linear DNA was PCR amplified from gDNA of the parent strain and purified plasmids, and linear DNA was digested with XbaI and PstI. After purification of products, samples were ligated using T4 ligase (NEB). Successfully ligated plasmids were transformed into DH5α cells for propagation using chemically competent cells and into the knockout strain Δ*gmlABC* via electroporation. Complementation plasmids were confirmed via sequencing of amplicons generated via PCR using check primers and visualized on a 1.5% agarose gel with Sybr Safe DNA gel stain (ThermoFisher).

### Western blots

OD_600_-normalized whole-cell lysates of each strain were separated on 4–20% Mini-PROTEAN precast polyacrylamide gels (BioRad). Samples were transferred to nitrocellulose membranes (BioRad) and blocked in LI-COR blocking buffer for 1 h. Membranes were incubated with primary antibody at 1:1,000 for 1 h and washed with TBS 0.1% vol/vol Tween-20 (TBST). After washing, membranes were incubated with secondary antibody for 30 min, washed with TBST, and imaged using an Odyssey Infrared Imaging System (LI-COR Biosciences). Primary polyclonal antibodies to O2α and O2β were a generous gift from Prof. Chris Whitfield (4) (University of Guelph). Secondary antibody was LiCor 800CW goat anti-rabbit at 1:10,000 dilution.

Type 1 and type 3 fimbriae were also measured using western blotting. Samples were separated on 15% polyacrylamide gels and transferred to PVDF membranes (BioRad). Membranes were treated as described above and stained with rabbit anti-type 1 pilus antibody 1:2,000 (Biosynth) for type I quantification and chicken anti-MrkA 1:10,000 (Biosynth) for type 3 quantification. Type I pilus antibody was produced by immunizing rabbits with soluble pilus protein, and type 3 by immunizing chickens with a purified MrkA peptide; both procedures were performed by Biosynth. Mouse anti-GAPDH (ThermoFisher) was used as a standard. Membranes were stained with anti-mouse HRP 1:5,000 (Biolegend), anti-rabbit HRP 1:10,000 (Biolegend), and anti-chicken IgY HRP 1:10,000 antibodies (Jackson Immuno Research). Membranes were imaged on a chemiluminescence reader (BioRad). Immunoblots were analyzed using ImageJ software, and quantification was normalized to GAPDH. Statistical analyses were performed using GraphPad Prism 10.

### Glucuronic acid quantification

Capsule polysaccharide was quantified using a glucuronic acid assay as previously described (9). Bacteria were grown statically overnight and resuspended in sterile PBS to a normalized OD_600_. Normalized culture was mixed with 1% Zwittergent 3-14 in 100 mM citric acid in triplicate and incubated at 50°C. After heating and centrifugation, samples were mixed with ethanol at 4°C. Following precipitation, samples were cleared by centrifugation, and pellets were dissolved in sterile water containing 12.5 mM tetraborate in concentrated sulfuric acid. Samples were boiled and mixed with 0.15% 3-hydroxydiphenol in 0.5% NaOH. Absorbance was measured at 520 nm on a microplate reader (BioTek). The uronic acid concentration was determined using a standard curve of glucuronic acid. Significance was determined using an unpaired *t*-test with *P* < 0.05. Statistical analyses were performed using GraphPad Prism version 10.

### Hypermucoviscosity assay

Hypermucoviscosity was determined using a centrifugation assay as previously described (14). Bacteria were grown statically overnight, pelleted, and resuspended in sterile PBS to a normalized OD_600_. Normalized cultures were centrifuged at 500 × *g* for 5 min, and the OD_600_ of the supernatant was measured and normalized back to the starting value. Significance was determined using an unpaired *t*-test with *P* < 0.05. Statistical analyses were performed using GraphPad Prism version 10.

### Biofilm quantification

Bacterial biofilm was quantified using a modified crystal violet staining assay (15). Bacterial cultures were grown statically overnight. The following day, sub-cultures in fresh LB broth were grown until mid-log phase (OD_600_ ~0.6). Cultures were then diluted 30-fold into LB broth and seeded into 96-well plates in replicates of six. Plates were incubated for 48 h at 25°C. Non-adherent cells were removed by gentle pipetting, wells were washed and allowed to dry, and then biofilm was resuspended in crystal violet solution 0.1% wt/vol for 15 min. Crystal violet solution was removed, and wells were washed and allowed to dry. Absorbance was measured after solubilization in ethanol at 595 nm using a microplate reader (BioTek).

### Flow cytometry

Lungs were perfused and harvested at given timepoints and deposited into sterile PBS. Lungs were transferred to digestion media (2.5 mg/mL Collagenase D, 3% fetal bovine serum, RPMI media), minced, and incubated for 1 h at 37°C with shaking. Digested lungs were filtered through a cell strainer and washed with FACS buffer (PBS, 0.5% bovine serum albumin, 2 mM EDTA). Red blood cells were lysed with Pharm Lyse Buffer (BD Biosciences) and cleared by centrifugation at 300 × *g*. Cells were washed, resuspended in FACS buffer, blocked with Fc Block (BD Biosciences) for 10 min, and stained at 4°C with the following antibodies: CD11b-Alexa700 (BD 557960), CD11c-BV785 (Biolegend 117336), F4/80-BB700 (BD 746070), Ly6G-FITC (BD 551460), MHCII-PerCP-Cy5.5 (BD 562363), Ly6C-ApCCy7 (Biolegend 128025), SiglecF-AF647 (BD 562680), CD45-BV510 (Biolegend 103138), and CD80-BV421 (Biolegend 104725). After staining, cells were washed, fixed with 2% paraformaldehyde, and analyzed on a Cytek Aurora cytometer. Total cell counts per lung were calculated using Precision Count Beads (Biolegend). Gating and analyses were performed using FlowJo version 10.

### Human serum killing assay

Blood specimens were collected by venipuncture from healthy adult donors, as approved by the Washington University in St. Louis Institutional Review Board (IRB protocol 201708048). Bacterial cultures were grown overnight, and the following day were pelleted and resuspended to a normalized OD_600_ in sterile PBS. Twenty-five microliters of bacteria were mixed in a 96-well plate with 75 µL of pooled human serum and incubated for 1 h at 37°C with shaking. Serum was either active or inactivated by heating at 56°C for 30 min. Samples were serially diluted and plated to calculate percent survival as the ratio of colony-forming unit (CFU) output to input CFU.

### Mouse immunization and pulmonary infections

All murine studies were approved by the Institutional Animal Care and Use Committee at Washington University School of Medicine (approved protocol 23-0300). Mouse immunizations and pulmonary infections were carried out as previously described (9, 14). Briefly, five-week-old CD-1 female mice were injected subcutaneously with 100 µL of vaccine formulation on days 0, 14, and 28. The vaccination groups were EPA (a non-toxic form of Exoprotein A from *Pseudomonas aeruginosa*), O2α-EPA, and O2β-EPA. Vaccines were produced via bioconjugation as previously described (9). All vaccines were formulated with Alhydrogel 2% aluminum hydroxide (InvivoGen) at a 1:9 ratio in Tris Buffered Saline (TBS). All groups received 1 µg vaccine based on total polysaccharide content. Sera was collected on days 0, 14, 28, and 42 prior to immunization or challenge. On challenge, mice were anesthetized with isoflurane and inoculated with bacterial suspension via oropharyngeal aspiration. Challenge doses for all strains were either 10^7^ CFU/50 µL for sub-lethal challenge or 10^8^ CFU/50 µL for pulmonary challenge after immunization. After challenges, organs were harvested and homogenized with sterile beads and Bullet Blender. Organ homogenates were serially diluted and plated to enumerate CFU per organ. Blood samples were collected via submandibular vein. Each experiment was performed in duplicate with *n* = 10 mice per group. All graphs and statistics were generated using GraphPad Prism version 10.

### ELISAs

All enzyme-linked immunosorbent assays (ELISAs) were carried out as previously described (9). 96-well plates were coated with ~10^6^ bacteria or 5 µg/mL of conjugate vaccine per well. After coating, wells were blocked with 1% bovine serum albumin in PBS and washed with 0.05% PBS-Tween-20 (PBST). Sera from mice were diluted 1:100 and added in triplicate for 1 h at room temperature. After washing, wells received HRP-conjugated anti-mouse (1:5,000) for 1 h at room temperature. Wells were washed and developed using 3,3′,5,5′-tetramethylbenzidine (TMB) substrate (Biolegend) and stopped with 2N sulfuric acid. Absorbance was read at 450 nm on a microplate reader (BioTek). Total IgG concentration was determined using an IgG standard curve. All wells were normalized to blank wells coated and treated the same as samples without receiving primary antibody. Significance was determined using Mann-Whitney nonparametric tests with *P* < 0.05. Statistical analyses were performed using GraphPad Prism version 10.

## RESULTS

### Manipulation of the gmlABC locus

**Fig 1:**
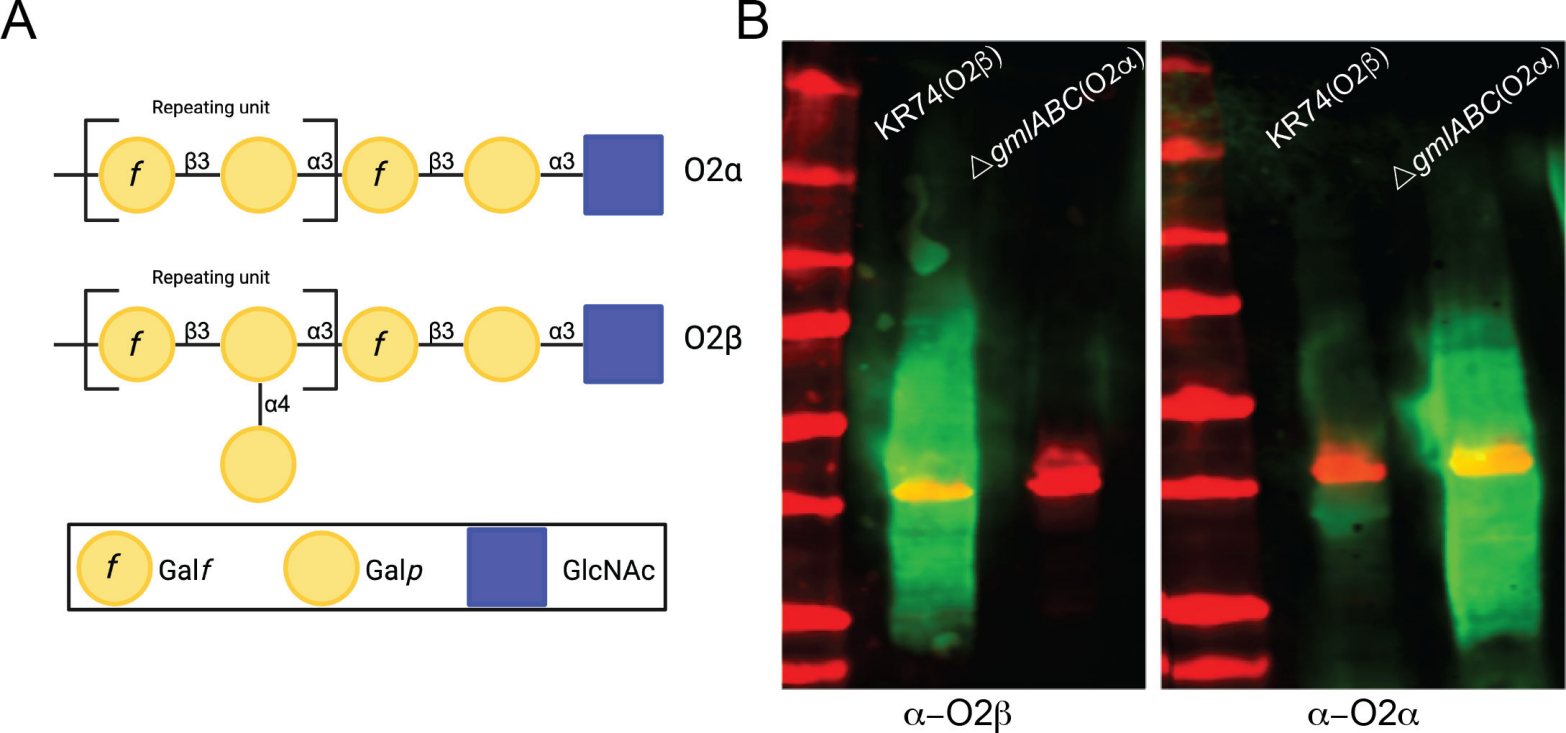
Construction of isogenic strains of the O2-antigen subtypes O2β and O2α. (**A**) Pictogram representation of the O2α and O2β subtypes. (**B**) KR74 parent strain was mutated by knocking out the *gmlABC* locus to transform the strain from O2β producing to O2α (Δ*gmlABC*). O-antigen production was verified via western blot of full cell lysates of the bacteria using antibodies against each subtype (4). Red band is RNA polymerase loading control.

The O2α and O2β subtypes of the O2 serogroup of *K. pneumoniae* OPS differ by only a single branched galactose residue (Fig. 1A). To assess fitness and immunologic differences imposed by this additional sugar, we deleted *gmlABC* in an O2β strain to create an isogenic O2α strain. The clinical *K. pneumoniae* isolate KR74 (K25: O2β) was collected from the BAL fluid of a 76-year-old female patient (11). Using a modified λ Red recombinase method, the *gmlABC* locus was deleted to successfully convert the isolate into the O2α-expressing strain (KR74Δ*gmlABC*). OPS production was confirmed via immunoblot analysis of whole-cell lysates using antibodies against O2α and O2β (4). The wild-type KR74 strain reacted strongly with the O2β antibody and exhibited slight O2α antibody cross-reactivity (Fig. 1B). The Δ*gmlABC* strain reacted only with the O2α antibody, confirming successful modification of an O2β strain to an O2α strain.

**Fig 2:**
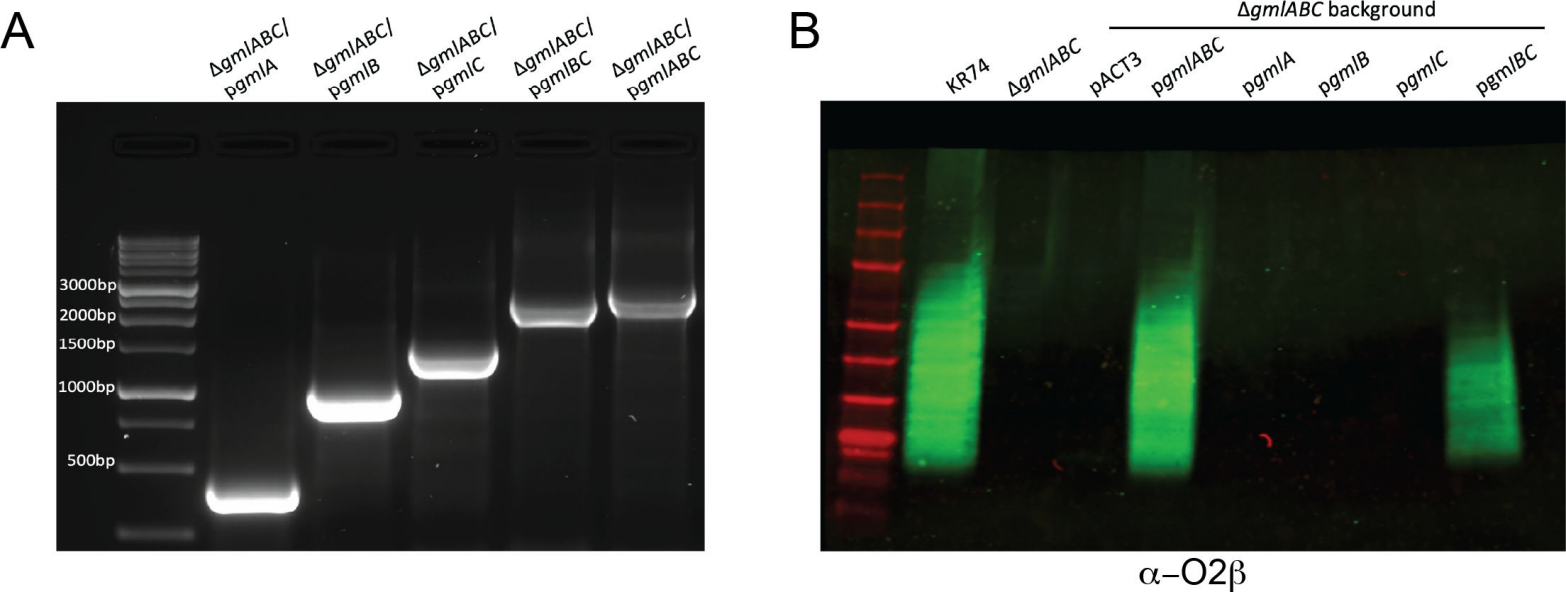
*gmlABC* complementation in *Klebsiella*. (**A**) Each individual gene*—gmlA*, *gmlB*, and *gmlC—*along with *gmlBC* and *gmlABC,* was cloned into pACT3 expression plasmid and electroporated into the Δ*gmlABC* strain. Complementation constructs were verified by PCR using check primers to amplify the expected fragments as displayed on the agarose gel. (**B**) Whole-cell lysates of the Δ*gmlABC* strain expressing each construct were probed via immunoblot with α-O2β antibody to test O-antigen expression and determine which constructs convert the strain to O2β expression.

While it is known that the *gmlABC* locus is responsible for conversion of an O2α strain to an O2β strain, the exact contributions of each gene in this operon are not defined; the functions of each gene have been proposed based on similar glycosylation systems in other organisms (4). To discover which *gml* gene(s) were responsible for addition of the galactose distinguishing O2α from O2β, we cloned each gene individually in an IPTG-inducible plasmid (pACT3) (9), as well as *gmlBC* and the full cassette *gmlABC* (Fig. 2A). Each construct was transformed via electroporation into KR74Δ*gmlABC*, and OPS reactivity was assessed via immunoblot. Probing with O2β antibody demonstrated reactivity with KR74 and the complete complement Δ*gmlABC*/p*gmlABC*, as expected (Fig. 2B). We did not observe an O2β signal upon complementation with any single gene. Interestingly, we observed O2β reactivity using our *gmlBC* complement (Fig. 2B), indicating these two genes are sufficient to make the O2β subtype.

### Virulence factor assessment of O2a and O2β strains

Upon verification of the isogenic O2α and O2β strains, we assessed if there were any differences in expression of recognized virulence factors or selected phenotypes between these two isolates (Fig. S1). We did not observe any significant differences in capsule production, hypermucoviscosity, biofilm formation, or production of type 1 and type 3 pili. Additionally, we tested for differences in pathogenic fitness of the two subtypes in an *in vivo* murine model of pneumonia. Female CD-1 outbred mice were challenged via pulmonary aspiration with a sublethal dose of KR74 (O2β) or Δ*gmlABC* (O2α). At 6 and 24 h post-infection, blood samples were taken to stain for innate immune cell markers. There were no significant differences between KR74 (O2β) and Δ*gmlABC* (O2α) in the numbers of macrophages, alveolar macrophages, dendritic cells, neutrophils, or eosinophils in peripheral blood (Fig. S2A through F). Further, there was no difference in lung bacterial burden between the two isolates at 6 or 24 h post-infection (Fig. S2G). These data indicate that the isogenic O2α and O2β strains behave similarly at early time points in the lung.

### Resistance to serum killing in O2β strains

**Fig 3:**
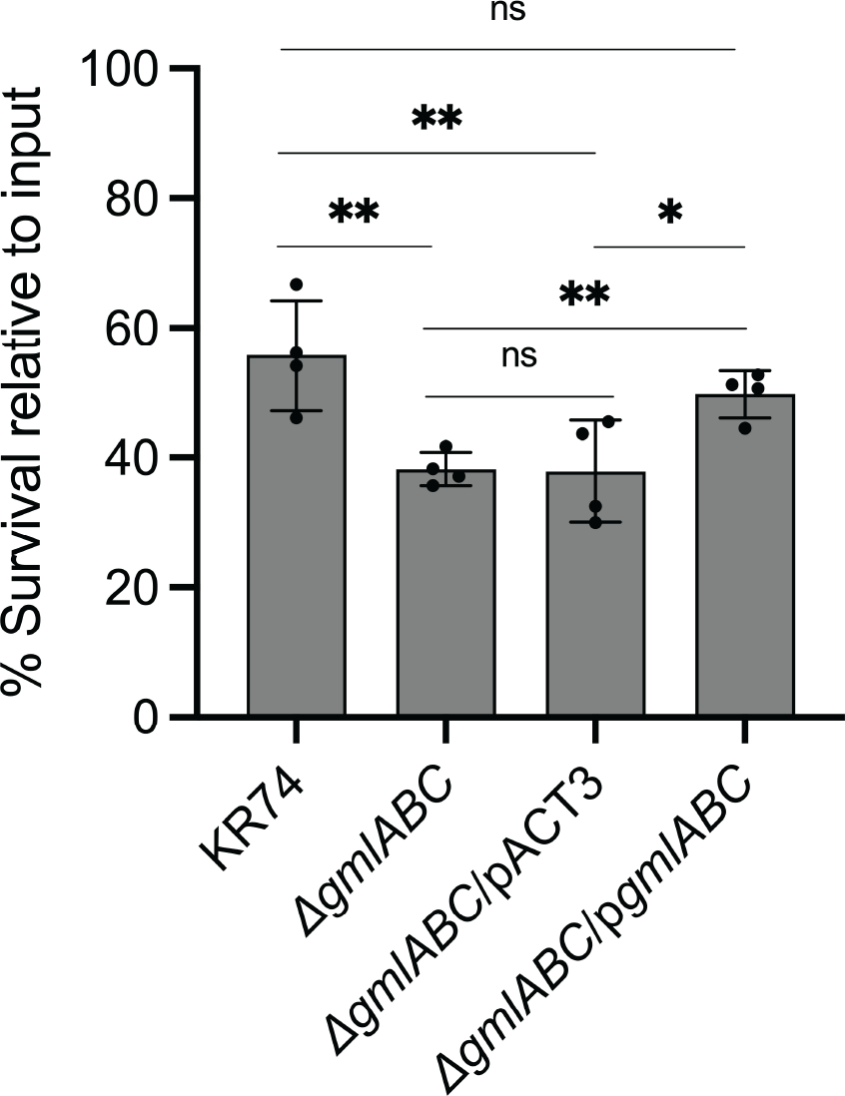
Strain survival in normal human serum. Strains were incubated in 75% normal human serum for 1 h, serially diluted to enumerated colonies, and counted. Percent survival was determined as output CFU compared to input CFU. Replicates from two independent experiments are shown. Statistics were performed using unpaired *t*-test. **P < 0.005, *P < 0.05; ns, not significant.

As it has been suggested that there may be increased resistance to serum-mediated killing in O2β strains compared to O2α (6), we sought to test this using our otherwise isogenic strains. Wild-type KR74 (O2β), the isogenic knockout (Δ*gmlABC*; O2α), empty vector control (Δ*gmlABC*/pACT3; O2α), and complemented strain (Δ*gmlABC*/p*gmlABC*; O2β) were incubated in pooled normal human serum at 37°C for 1 h. Importantly, human serum was taken from multiple donors with no known exposure to *K. pneumoniae*. None of the donors in the pooled serum exhibited significant titers to the strains used. The O2α-expressing strains had significantly lower survival in human serum compared to the O2β-expressing strains (Fig. 3), and the Δ*gmlABC* phenotype was ameliorated in the complemented strain, indicating that the addition of the single branched galactose residue in the O2β structure affords a modest increase in serum resistance.

### Differences in adaptive immune response between O2α and O2β subtypes

**Fig 4:**
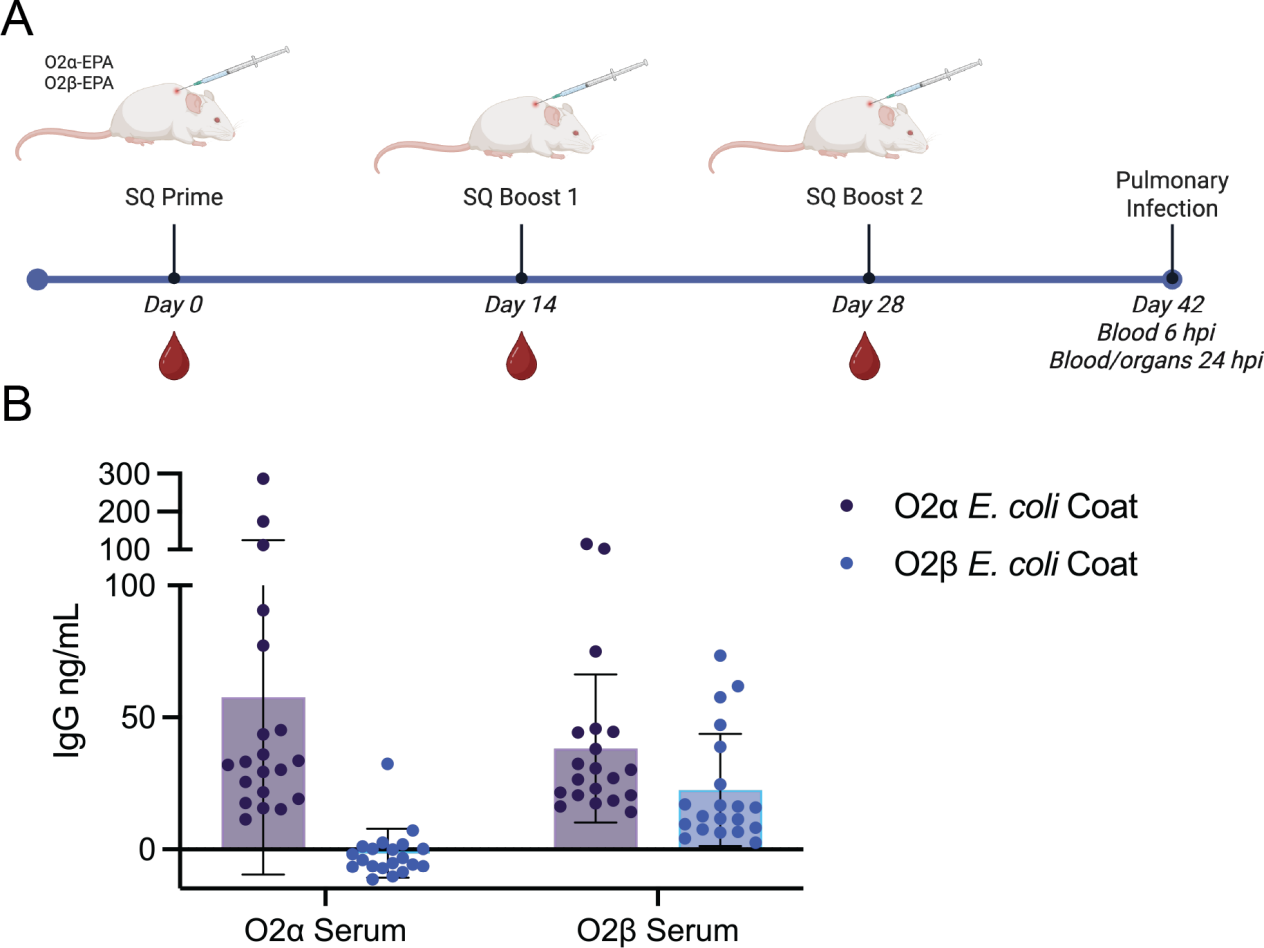
Antibody production after immunization with O2α and O2β conjugate vaccines. (**A**) CD-1 female mice were immunized with either O2α-EPA or O2β-EPA at time points depicted in the immunization schematic. Blood samples were taken at indicated days. (**B**) Serum IgG at day 42 was measured via ELISA on plates coated with glycoengineered *E. coli* producing each O2 subtype. The x-axis indicates the immunization group. ELISA depicts serum concentrations at a 1:100 dilution.

We next sought to explore adaptive immune responses to these antigens. We utilized bioconjugate vaccines developed against O2β-EPA and O2α-EPA, as previously described (9). These vaccines included EPA as a carrier protein and alum as an adjuvant. CD-1 female mice were immunized via subcutaneous injection as depicted (Fig. 4A). Fourteen days after the final immunization (i.e., day 42), serum was tested specifically for O2α and O2β antibodies using glycoengineered strains of *E. coli* as previously described (9). These glycoengineered strains lack their own capsule or O-antigen and have been transformed to produce the *K. pneumoniae* OPS of interest (either O2α or O2β). Importantly, the polysaccharide structures were validated by 2D-NMR (9), and the polysaccharides produced are identical to those produced by *K. pneumoniae*. Mice immunized with the O2α conjugate vaccine generated antibodies against only O2α, but not O2β (Fig. 4B). On the other hand, mice immunized with the O2β conjugate generated antibodies reacting to O2α and O2β, demonstrating cross-reactivity of the antibodies generated by the O2β antigen (Fig. 4B).

We also investigated the antibody response generated after pulmonary infection (live bacteria vaccination) of naïve female CD-1 mice with KR74 (O2β) or KR74Δ*gmlABC* (O2α). Mice were infected with a non-lethal dose of bacteria, and serum was collected 28 days after inoculation and was tested for O2α and O2β antibodies. Mice infected with KR74 generated antibodies reactive to both O2α and O2β (Fig. S3). Surprisingly, we did not observe any measurable IgG against either O2α or O2β in mice infected with the O2α strain KR74Δ*gmlABC* (Fig. S3). Together, these data indicate a difference in antibodies elicited by these two subtypes: O2β antigen generates antibodies with strong O2β reactivity and moderate O2α cross-reactivity. Further, O2β may be more immunogenic, with the O2β vaccine and strain eliciting greater antibody production overall.

### Vaccination lowers the burden against O2β but not O2α

**Fig 5:**
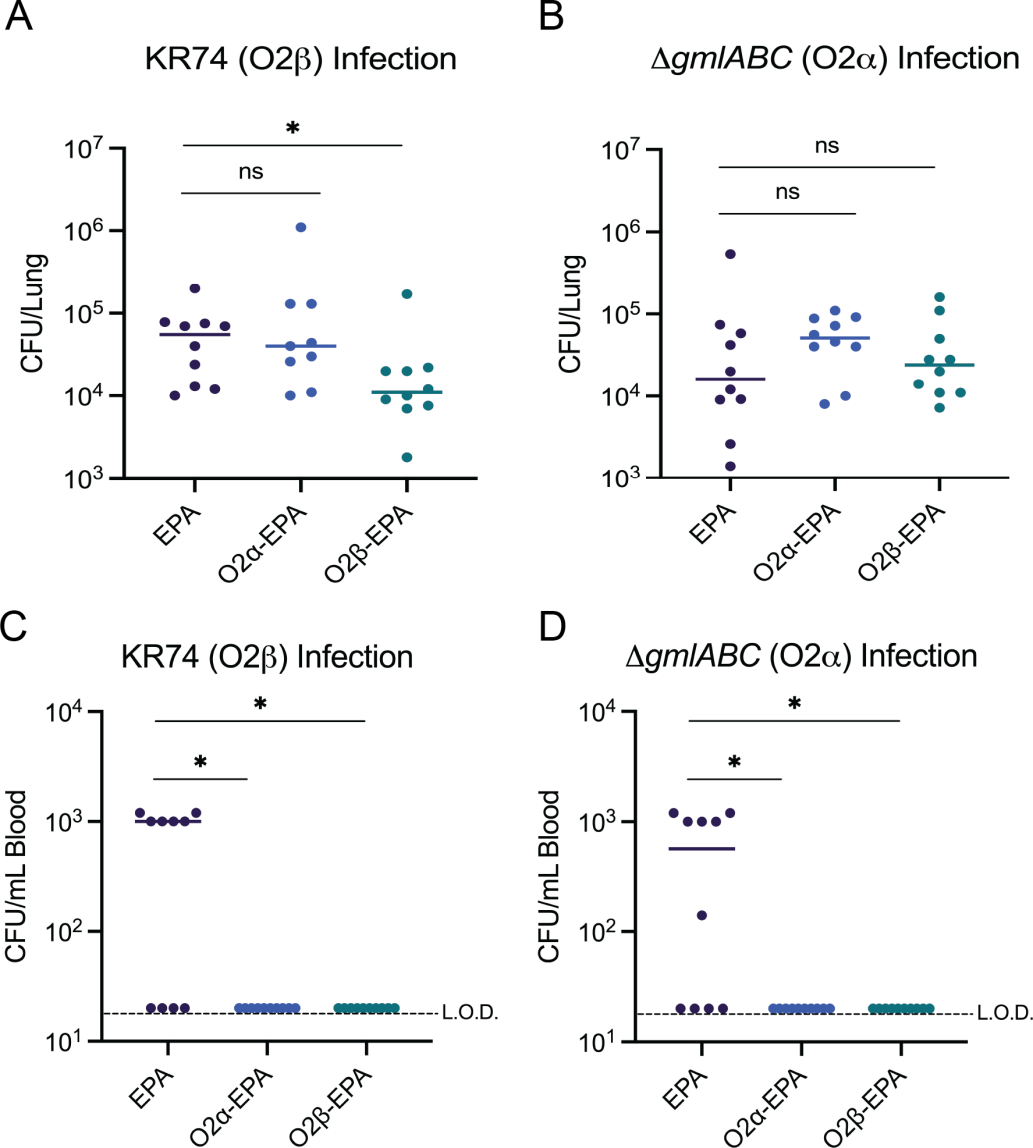
Organ titers after infection with KR74 and *DgmlABC*. Female CD-1 mice were immunized with either EPA, O2α-EPA, or O2β-EPA and at day 42 were infected with either (**A**) KR74 or (**B**) *DgmlABC* isolates. At 24 h post-infection, lungs were harvested, homogenized, and CFU enumerated. At 6 h post-infection, blood was drawn from (**C**) KR74-infected mice or (**D**) *DgmlABC*-infected mice, and CFU were enumerated. Statistics were performed using Mann-Whitney. *P < 0.05; ns, not significant. L.O.D. = limit of detection of 20 CFU.

We next tested whether immunization against O2α or O2β would provide homologous or cross-reactive protection against pulmonary challenge with *K. pneumoniae*. Three groups of mice, EPA, O2α-EPA, and O2β-EPA, were immunized and challenged via aspiration at day 42 with either KR74 or KR74Δ*gmlABC*. Blood was collected 6 h post-infection, and at 24 h post-infection, mice were euthanized, and the lungs were harvested. In mice challenged with KR74 (O2β), there was no difference in lung bacterial burden between EPA control mice and mice immunized with O2α-EPA; in contrast, we observed a significant decrease in bacterial burden in mice immunized with O2β-EPA (Fig. 5A). Interestingly, neither vaccination resulted in an observed decrease in bacterial burden upon infection with KR74Δ*gmlABC* (O2α) (Fig. 5B). Of note, both vaccines appeared to inhibit dissemination from the lung, as mice immunized with either O2α-EPA or O2β-EPA did not exhibit bacteremia, while 60% of control (EPA-vaccinated) mice did (Fig. 5C and D). These data suggest that while both vaccines may prevent dissemination from the lung, only the O2β vaccine led to decreased lung bacterial burden after infection with a matched strain.

## DISCUSSION

O2α and O2β subtypes of the O2 OPS serogroup of *K. pneumoniae* structurally differ by a single galactose residue in each repeating unit. The implications of this seemingly small difference were the focus of the present investigations. It has been shown that antibiotic-resistant strains of *K. pneumoniae* more often produce O2 OPS types, specifically O2β. While others have suggested fitness or immune differences between these two subtypes utilizing *in trans* expression of *gmlABC* or different strains entirely (4, 6), such differences have never been examined using isogenic strains. Further, adaptive responses to these O-antigen subtypes have not been thoroughly examined but could inform future vaccine design. The present work adds to our knowledge of these antigens by more closely examining the adaptive immune response from a natural infection with *K. pneumoniae* isolates producing either of these O-antigen subtypes to inform host antibody production. Additionally, we explore vaccines against these two O-antigen subtypes and their ability to protect from infections caused by O2-producing isolates of *K. pneumoniae*.

Our complementation studies indicate that the ability to add the α1,4Gal*p* sugar to the O2α backbone requires only the *gmlB* and *gmlC* genes, while *gmlA* may be dispensable. The role of each gene has previously been hypothesized (3). GmlB is a polyprenyl phosphosugar synthetase, and GmlC is a glycosyltransferase that adds the side group (3, 4). GmlA is thought to be a multi-antimicrobial extrusion family flippase that exports the undecaprenyl-phosphate–linked galactose residue, und-P-Gal*p*, to the periplasm. Based on our analysis, it appears that *gmlBC* largely restores production of the O2β phenotype, but it appears that restoration may be slightly decreased compared to wild-type expression. GmlA may be required for optimal production of longer branches of the O-antigen. Additionally, while we do not have direct supportive evidence, it is possible that a different Wzx-like flippase encoded by KR74 may promiscuously export und-P-Gal*p*, enabling galactosylation of the nascent O2α polymer.

Our results are consistent with other experiments leveraging different O2α isolates and *in trans* expression of genes required for production of O2β. First, it was shown that when O2β was produced via plasmid complementation with *gmlABC*, this strain had increased serum resistance compared to the parent O2α strain (6). Similarly, in our isogenic strains, the parent O2β exhibited increased serum survival compared to the Δ*gmlABC* O2α strain. Importantly, we were also able to complement the O2β phenotype on a plasmid and saw restoration of serum resistance. Secondly, the previous group also observed no differences in bacterial burden after pulmonary or bloodstream infection with either subtype (6). While we only performed a pulmonary challenge, we also did not observe a significant difference in virulence between the two subtypes *in vivo*. Thus, while these isolates do not exhibit differences in pulmonary virulence, an enhanced ability of O2β strains to survive in human serum may represent an evolutionary advantage of O2β strains.

It has been demonstrated through molecular dynamics and modeling studies that the addition of the α1,4Gal*p* side chain has substantial effects on polysaccharide conformation (16), specifically favoring a predominantly extended state of the OPS chain. It is hypothesized that this extended form could influence the amount of the OPS barrier on the bacterial surface which might promote resistance to serum (3). Further, an extended form of OPS in O2β compared to O2α might alter antigen exposure, which could explain the differences we see in antibody binding between the subtypes. Importantly, these data are consistent with the antibody binding profile we have previously observed after vaccination (9). It appears that after immunization with our conjugate vaccines, O2α antibodies recognize only O2α strains, but O2β antibodies have some level of recognition for both O2β and O2α. Aligned with our findings, a recent study of antibodies to *K. pneumoniae* found that two antibodies, UKpn6 and UKpn7, were able to recognize O2α strains but not O2β isolates. The authors hypothesized that these antibodies bind an epitope that may be shielded in O2β isolates but not in O2α (17).

These data highlight the importance of studying the immune response to these two subtypes to better inform immunotherapies, such as vaccines, based on predominance of circulating strains. We have demonstrated that O2α antibodies do not cross-react with or protect from O2β-producing isolates of *K. pneumoniae*, nor do they protect against O2α-producing strains. However, O2β antibodies may be able to bind both O2α- and O2β-producing strains and provide partial protection from pulmonary infection with a matched strain. We conclude that O2β may be a more suitable subtype for inclusion in candidate vaccines against this troublesome pathogen.

## SUPPLEMENTAL MATERIAL

Supplemental material iai.00538-25-s0001.pdf Fig. S1 to S3; Table S1.

ASM does not own the copyrights to Supplemental Material that may be linked to, or accessed through, an article. The authors have granted ASM a non-exclusive, world-wide license to publish the Supplemental Material files. Please contact the corresponding author directly for reuse.
