# Supplementary material for: Differential virulence and immune recognition of Klebsiella pneumoniae O-antigen subtypes O2α and O2β
Source: Infect Immun. 2025 Nov 28;94(1):e00538-25. doi: 10.1128/iai.00538-25 (PMC12797948; doi:10.1128/iai.00538-25)
Supplement: Supplemental material — Fig. S1 to S3; Table S1. [file iai.00538-25-s0001.pdf]

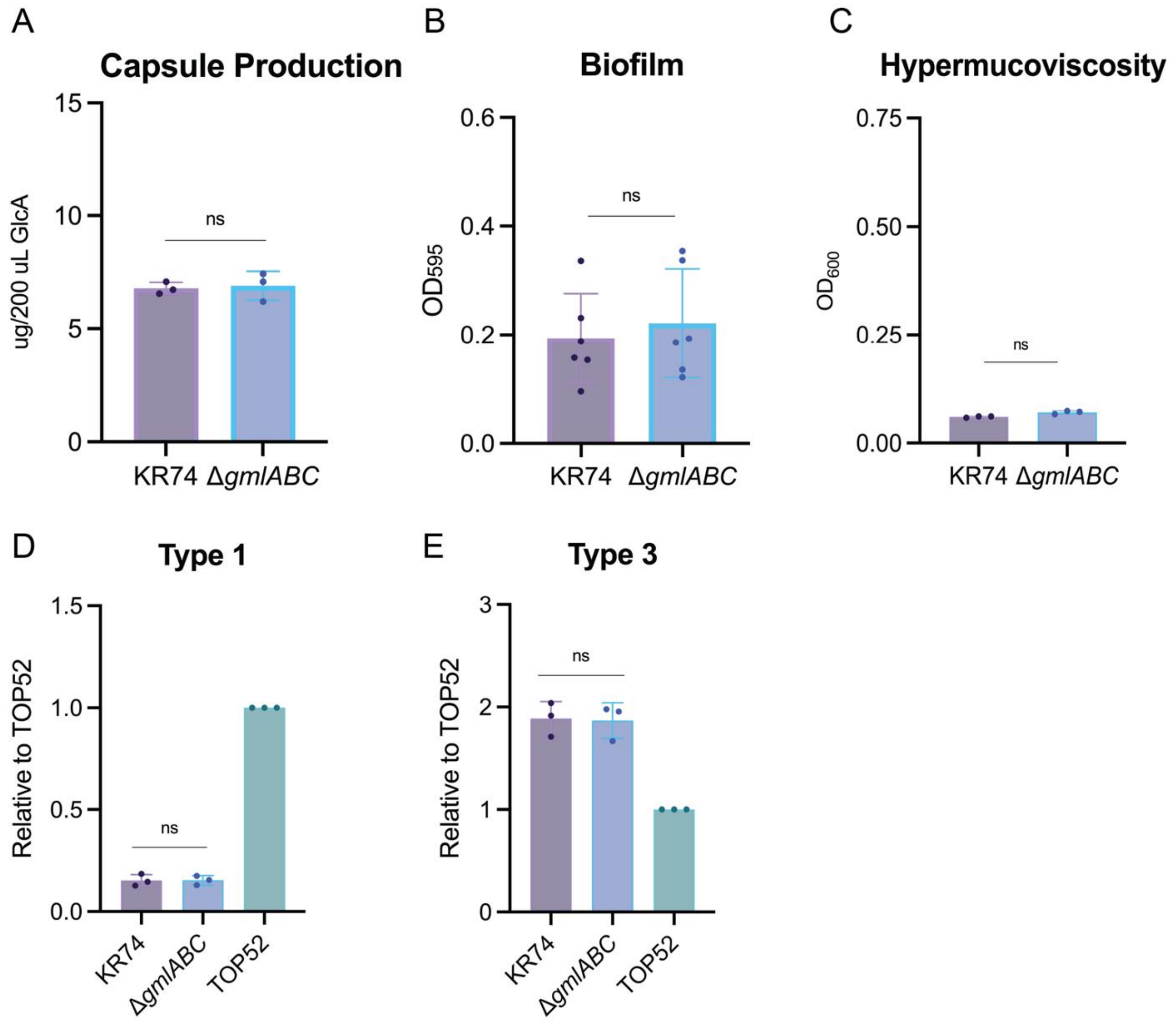

Supplemental Figure 1: Phenotypic expression of virulence factors. A) Glucuronic acid quantification of KR74 and *ΔgmlABC*. B) Biofilm formation was measured via crystal violet staining. C) Hypermucoviscosity was measured via centrifugation assay. Type I (D) and type 3 (E) pili expression were measured via western blot and quantified using ImageJ software. Statistics were performed using unpaired t-test. ns=not significant.

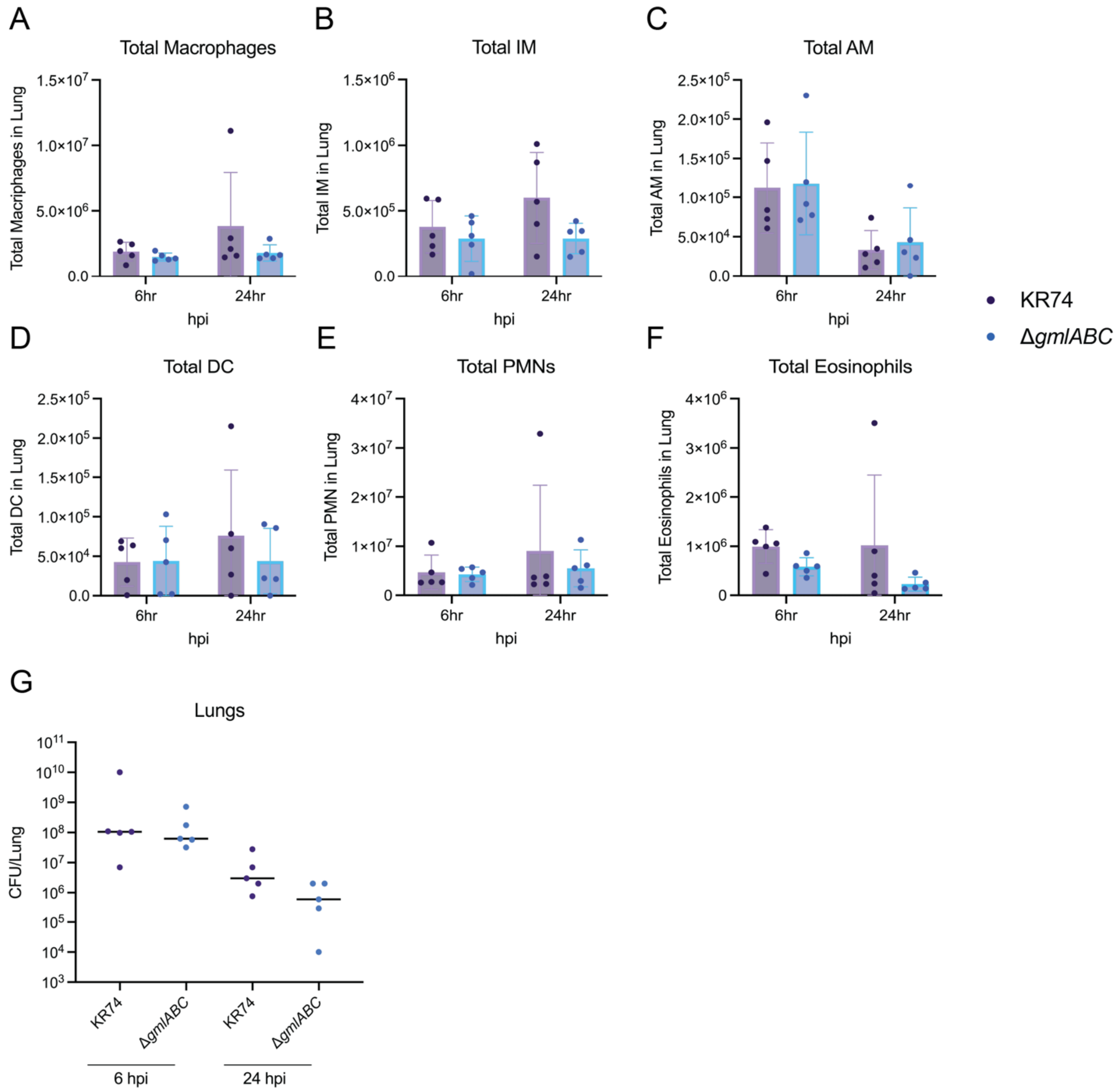

Supplemental Figure 2: Flow cytometry analysis of innate immune cells after sub-lethal infection. CD-1 female mice were administered ~10^7 CFU of KR74 or *ΔgmlABC*. Six or 24 h post infection, lungs were harvested and stained for A) total macrophages, B) total interstitial macrophages, C) total alveolar macrophages, D) total dendritic cells, E) total polymorphonuclear lymphocytes, and F) total eosinophils. G) Bacterial CFU in lung homogenates were enumerated 6 and 24 h post infection. Statistics were performed using Mann-Whitney U tests, as not all data were normally distributed.

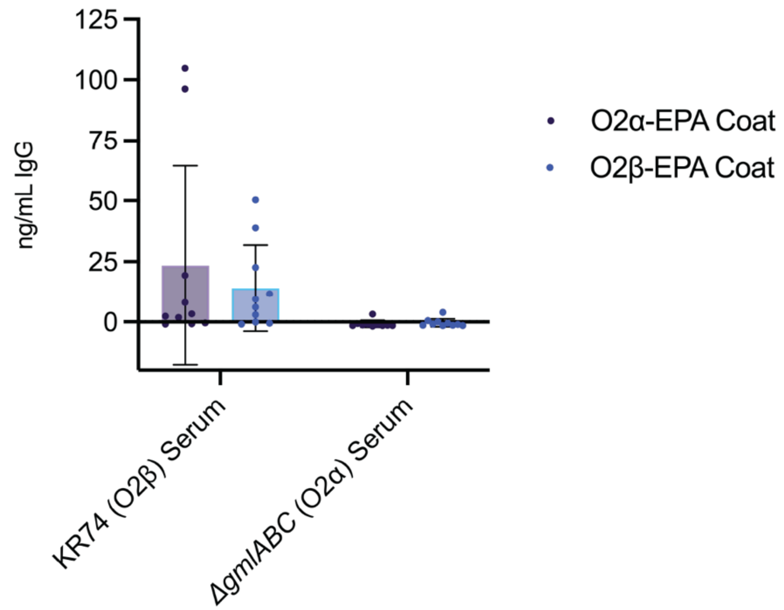

Supplemental Figure 3: Antibody titers after pulmonary infection with KR74 or *ΔgmlABC*. Female CD-1 mice were infected with 10^7 CFU of KR74 or *ΔgmlABC*. Twenty-eight days post infection, sera were collected and tested for O-antigen-specific IgG on ELISA plates coated with O2α or O2β conjugate vaccines. ELISA depicts serum concentrations at a 1:100 dilution.

Supplemental Table 1: Primers used in this study

Primer Name	Sequence
gmlA For	GCATGTCTAGATATATAATGCCAAGTTCAGGCCCATTATGGCAACTA (with XbaI site)
gmlA Rev	CTTGACTGCAGGGTTGTCATTTCATCGCCCAGAACTTGTTTAAAATAA (with PstI site)
gmlB For	GCATGTCTAGAGATGAAATGACAACCTCAACTGATATAAAAAGCACTCC (with XbaI site)
gmlB Rev	CTTGACTGCAGTTGCATGTTATTTTCCATTGCTATCATTACCTACGCT (with PstI site)
gmlC For	GCATGTCTAGAAATAACATGCAAAATCTGATCAATCCTTTAGCAGAGGG (with XbaI site)
gmlC Rev	CTTGACTGCAGATTACTAATAATTTATCGTTGACCTTCGCATTGCATCTGA (with PstI site)
gmlABC For RR Method	AATTGGAACGCTAGCGGAGTATATAATGCCAAGTTCAGTGTAGGCTGGAGCTGCTTC
gmlABC Rev RR Method	AAATGAGGGCGGCATAGAAATTACTAATAATTTATCGTTGCATATGAATATCCTCCTTAG
Check primer For	CCCTTCACGCAGCACTCGAA
Check primer Rev	GTCTGTTCAGACAAAGTTTC
